# Sevoflurane protects against cerebral ischemia/reperfusion injury via microrna-30c-5p modulating homeodomain-interacting protein kinase 1

**DOI:** 10.1080/21655979.2021.1999551

**Published:** 2021-12-09

**Authors:** Guoning Su, Yan Qu, Gang Li, Min Deng

**Affiliations:** Department of Anesthesia, Affiliated Hospital of Yunnan University, KunMing City, China

**Keywords:** Cerebral ischemia-reperfusion injury, Sevoflurane, MicroRNA-30c-5p, Homeodomain-interacting protein kinase 1

## Abstract

Sevoflurane (SEV) has been reported to be an effective neuroprotective agent for cerebral ischemia/reperfusion injury (CIRI). However, the precise molecular mechanisms of Sev preconditioning in CIRI remain largely unknown. Therefore, CIRI model was established via middle cerebral artery occlusion method. SEV was applied before modeling. after successful modeling, lentivirus was injected into the lateral ventricle of the brain. Neurological impairment score was performed in each group, and histopathologic condition, infarct volume, apoptosis, inflammation, oxidative stress, microRNA (miR)-30 c-5p and homeodomain-interacting protein kinase 1 (HIPK1) were detected. Mouse hippocampal neuronal cell line HT22 cells were pretreated with SEV, and the *in vitro* model was stimulated via oxygen-glucose deprivation and reoxygenation. The corresponding plasmids were transfected, and the cell growth was detected, including inflammation and oxidative stress, etc. The targeting of miR-30 c-5p with HIPK1 was examined. The results clarified that reduced miR-30 c-5p and elevated HIPK1 were manifested in CIRI. SEV could improve CIRI and modulate the miR-30 c-5p-HIPK1 axis *in vitro* and *in vivo*, and miR-30 c-5p could target HIPK1. Depressed miR-30 c-5p could eliminate the protection of SEV *in vitro* and *in vivo*. Repression of HIPK1 reversed the effect of reduced miR-30 c-5p on CIRI. Therefore, it is concluded that SEV is available to depress CIRI via targeting HIPK1 through upregulated miR-30 c-5p.

## 1. Introduction

Stroke is the 2^nd^ main reason of death after coronary artery disease [1], and timely thrombolysis and quick and effective reconstruction of microvascular collateral circulation to regain blood reperfusion in the penumbra and ischemic areas during treatment are the best approaches for the therapy of cerebral ischemia [2], nevertheless, blood flow after ischemia and repetency may cause ischemia/reperfusion injury (i/r), further aggravating brain tissue damage [3].

Some drugs are available for organ protection, named as pharmacological preconditioning. plenty of studies have assured that volatile anesthesia preconditioning mimics organ protection in all kinds of tissues and organs [4–6]. Sevoflurane (sev) is a frequently applied inhaled anesthetic, analogous to isoflurane and desflurane [7] and in sorts of i/r experimental animal models, sev pre- and post-treatment have been manifested to ameliorate damage [8–10]. Nevertheless, the underlying mechanism of the safeguard of sev preconditioning on cerebral ischemia/reperfusion injury (ciri) is still required to be further discovered and explored.

Accumulated studies have assured the crucial micrornas (mirnas) and their target genes in controlling i/r damage in animal or cell models [11,12]. Meanwhile, mirnas can also influence organ damage after sev treatment via modulating associated pathways. for instance, sev is available to modulate insulin-like growth factor 1 expression through mir-214 to stimulate liver injury [13], and sev can decline neuroinflammation caused by ciri via up-regulating mir-203 with targeting myd88 after treatment [14]. MiR-30 c-5p is demonstrated as a vital modulator of some myocardial abnormalities and is usually applied as a therapeutic target to alleviate myocardial ischemia-reperfusion injury [15–17]. Nevertheless, few studies on mir-30 c-5p in ciri are manifested.

Homeodomain-interacting protein kinase 1 (hipk1) is a modulator of homologous domain transcription factors modulating various cell biological processes associated with inflammation and anti-stress responses [18]. A study has affirmed that snhg14 retrains lipopolysaccharide-stimulated proliferation and autophagy of HK-2 cells through mir-495-3p/hipk1 axis, and accelerates apoptosis and the generation of inflammatory factors [19]. Nevertheless, the mechanism of hipk1 in ciri remains ambiguous.

The study was to explore the beneficial function of sev on ciri in rats. The molecular mechanism of sev in ciri was further studied, with emphasizing on the regulation of mir-30 c-5p/hipk1 axis. It was hypothesized that sev protected ciri via modulating hipk1 expression through mir-30 c-5p. Taken together, in our results novel insights will be provided on the mitigation of sev on ciri.

## 2. Materials and Methods

### 2.1. Establishment of CIRI model and grouping

A total of 60 male c57bl/6 rats (experimental animal center, chinese academy of medical sciences, beijing, china), weighing 250–280 g, was raised in a cage with relative humidity of 50%-60%, at room temperature: 20°C-23°C, with 12-h light cycle, and free to gain food and water. the rats were randomly assigned into 5 groups. Middle cerebral artery occlusion (mcao) was applied for establishment of ciri model as set forth. the rats were anesthetized with 10% chloral hydrate (350 mg/kg), then the midline skin was cut open to expose the external carotid artery (eca) and the internal carotid artery (ica), which were then ligated. the heparinized endovascular wire (0.28 mm, beijing cinontech co., ltd, beijing, china) was inserted to ica via eca to block the origin of middle cerebral artery. After sealing for 2 h, slowly pulling out the suture was conducted. Then reperfusion was performed, and the rats were placed in an intensive care incubator for 24-h restoration. The sham rats were conducted with same operation without inserting sutures. Before mcao, the rats were pretreated *in vivo* with 2.5% sev (baxter international inc., deerfield, il, usa) inhalational administration system in 33% oxygen and air mixture 30 min a day for 4 consecutive days [20]. Two days before MCAO, 5 μL miR-30 c-5p antagomir, negative control (NC) and si-HIPK1 lentivirus (10^9^ infected units/ml) (genen pharmaceutical co., ltd., shanghai, china) [21] were implemented with lateral ventricle injection. directional injection is carried out in reference to a previous scheme [22]: all mice were anesthetized by 2%-2.5% isoflurane ventilation and placed in a stereotaxic framework. the animals were placed in a stereotaxic framework, and 26 g cerebral infusion cannula was introduced into the left ventricle, with coordinates as follows: anterior fontanel: 0.58 mm; dorsal abdomen: 2.1 mm; transverse: 1.2 mm. after surgery, the mice were kept at 37°C until they fully regained consciousness, and then were conducted with neurological function score. three days later [23], the rats were euthanized. then the whole brain tissue was harvested. all animal experiments were carried out in line with the standard procedures permitted via the ethics committee of affiliated hospital of yunnan university [24]. Treatment and all analysis were performed via adopting the blinding method.

### 2.2. Neurological deficit score

Obeying former literature [25], neurological impairment score: 0, with no symptoms of neurological impairment; 1, the rat’s contralateral forelimb is not available to be fully extended; 2, turning the body to the hemiplegic side while walking; 3, lean to the hemiplegic side while walking; 4, the rats were unable to self-induce unconsciousness. Neurological deficits were scored 24 h after reperfusion.

### 2.3. 2, 3, 5-Triphenyltetrazolium chloride (TTC) Staining

The fresh brain sections with 1 mm thickness were soaked in 2% ttc-phosphate buffered saline (PBS) solution for 20 min in darkness at room temperature. After a brief wash with PBS, the sections were fixed in 4% paraformaldehyde (Sigma, St. Louis, MO) for 30 min at room temperature. These sections were shot via a digital camera. ImageJ 1.51 was used to measure each brain section and the infarct area (unstained area). The infarct volume was calculated as (infarct area/total brain area)100% [26].

### 2.4. Hematoxylin-eosin (HE) staining

The rat brain tissue was immersed in 4% paraformaldehyde for preparation of paraffin sections, cut into 4 μm, roasted, de-waxed and hydrated regularly. then the paraffin sections were stained with he solution, after which, the damaged neurons manifested nuclear constricting, cell edema, vacuolization and nucleus [27].

### 2.5. Terminal deoxynucleotidyl transferase-mediated dUTP-biotin nick end-labeling (TUNEL) staining

Tunel staining was conducted with the Apoptosis Kit (Roche, China) with the manufacturer’s instructions. Briefly, after dewaxing, the sections were incubated with protease K, membrane breakdown solution, tunel reagent, 3% hydrogen peroxide, and the conversion agent-pod, and developed via diaminobenzidine. tunel positive neurons around the damaged area (Olympus, Japan) were observed and counted under fluorescence microscope [28].

### 2.6. Cell culture and establishment of oxygen-glucose deprivation reoxygenation (Ogd/R) model in vitro

Mouse Hippocampal Neuronal Cell Line HT22 cells (American culture, Manassas, VA, USA), were cultured in dulbecco modified eagle medium (dmem) incubator consisting of 10% fetal bovine serum (FBS) and 100 u/ml penicillin and streptomycin. As set forth [29], in the oxygen-glucose deprivation (OGD) treatment, HT22 cells were first placed in a glucose-free hypoxic medium, then cultured in a hypoxic container at 37°C, with 95% N_2_/5% CO_2_ for 4 h. Then the cells were transferred to a complete DMEM containing 10% FBS, and cultured under 37°C with normoxia (95% air, 5% CO_2_) for 24 h. Then, the cells were introduced with miR-30 c-5p inhibitor, si-HIPK1 and its Nc (RiboBio) with 30 μL Lipofectamine 3000 reagent (Invitrogen Life Technologies, USA). The collection of cells was conducted for later experiments. After incubation for 24 h, the cells were subjected to subsequent experiments.

### 2.7. SEV pretreatment

The cells were placed in a sealed anesthetic chamber containing 2.5% SEV and 5% CO_2_/95% air, and immersed for 30 min, with 3 times’ repetition, and rinsed for 15 min after each exposure. Twenty-four h after SEV pretreatment, the cells were received with Ogd/R [20].

### 2.8. Detection of reactive oxygen species (ROS)

The 1 × 10^4^ HT22 cell suspension was gained and supplied with ROS probe dichlorofluorescin diacetate (DCFDA) (Beyotime, Shanghai, China) as set forth. After incubation, the cells were centrifuged at 300 *g* and re-suspended. Relative ROS levels were determined by Fluorescence-activated cell sorter (FACS) (FACScan; BD Biosciences inc., Franklin Lakes, New Jersey, USA) [30].

### 2.9. Enzyme Linked Immunosorbent assay (ELISA)

Measurement of interleukin (IL)-1β, tumor necrosis factor α (TNF-α) and IL-6 in OGD/R-treated HT22 cells and rat brain tissue was with elisa kits obeying manufacturer’s protocol. The content of malondialdehyde (MDA), glutathione peroxidase (Gsh-Px) and superoxide dismutase (SOD) were detected with the kit with the manufacturer’s instructions.

### 2.10. Cell counting kit 8 (CCK-8) analysis

CCK-8 (Cat#CK04-01; Dojindo, Kumamoto, Japan) reagents were mixed with cell medium with manufacturer’s instructions. the cells were then incubated. Absorbance was recorded at 450 nm applying a microplate reader (BioTek, Winooski, VT, USA).

### 2.11. Colony Formation Assay

HT22 cells were treated with trypsin. The 1 × 10^3^ cells were placed in 6-well plates and the colonies were stained with the staining solution composed of methanol (20%) and crystal violet (0.1%). The cells were counted and analyzed [31].

### 2.12. Transwell migration and invasion assay

Transwell inserts (Corning, Inc.) with 8 µm aperture was inserted into a 6-well plate. HT22 cells (1 × 10^5^ cells/well) were seeded into the upper chamber of transwell and cultured in serum-free medium (Gibco; Thermo Fisher Scientific, Inc.). for the assay of cell invasion, transwell’s upper chamber was covered with 50 µL Matrigel (BD Biosciences) at 37°C for 2 h. The complete medium supplemented with 10% FBS (Gibco; Thermo Fisher Scientific, Inc.) was added to transwell’s lower chamber. after culture for 24 h, the cells were fixed with 4% paraformaldehyde (Sigma Aldrich; Merck KGaA) at room temperature for 20 min, and stained at room temperature with 0.1% crystal violet (Sigma Aldrich; Merck KGaA) for 10 min. The number of migrating and invading cells was determined under a light microscope (zeiss AG) under five random fields (magnification, × 100) at 10 min [32].

### 2.13. Flow cytometry

Apoptotic cells were quantified via flow cytometry applying the Annexin V- fluoresceinisothiocyanat (FITC) Apoptosis Assay Kit (BD Biosciences, San Jose, CA) as per instructions. Simply put, the cells were harvested by trypsin detachment, seeded into 6-well plates at a density of 2.0 × 10^5^ cells per well, and re-suspended in a 200 uL binding buffer consisting of 10 μL Annexin V-FITC and 5 uL propidium iodide. Apoptosis cells were detected by flow cytometry (Annexin V positive) [33].

### 2.14. Reverse transcription quantitative polymerase chain reaction (RT-qPCR)

Extraction of total RNA was from tissues or cells via TRIzol reagents (Invitrogen, Waltham, MA, USA). Detection of the concentration and purity of RNA was via nano-droplet spectrophotometer. With the instructions, reverse transcription of 1 μg total RNA was via the PrimeScript RT kit (Promega, Madison, WI, USA) and synthesis of its complementary DNA (cDNA) was conducted. performance of RT-qPCR was via SYBR Premix Ex TaqTM kit (Takara, Otsu, Japan). The loading control of HIPK1 was glyceraldehyde-3-phosphate dehydrogenase (GAPDH), and that of miR-30 c-5p was U6. The primers were synthesized by RiboBio (Guangzhou, China) [34].

**Table:** 

Genes	Forward (5ʹ-3ʹ)	Reverse (5ʹ-3ʹ)
HIPK1	TCCCCATACTACGAGAAGGGT	ATGTCCCCACCCCTAGTACC
GAPDH	CATTCAAGACCGGACAGAGG	ACATACTGCACACCAGCATCACC
MiR-30 c-5p	GCGCGTGTAAACATCCTACACT	AGTGCAGGGTCCGAGGTATT
U6	GAAGCGCGGCCACGAG	AGTGCAGGGTCCGAGGTATT

### 2.15. Western Blot detection

Total protein was extracted from tissues or cells via radioimmunoprecipitation lysis buffer (Beyotime Biotechnology Co., Ltd., Shanghai, China). Measurement of protein concentration was via the bicinchoninic acid Protein Determination Kit (Beyotime Biotechnology). separation of the protein samples of 30 μg was via 10% sodium dodecyl sulfate polyacrylamide gel electrophoresis and blotted on polyvinylidene membrane (Millipore). After block with 5% skim milk, incubation of the membrane was with anti-HiPK1 (1: 1000; ab90103; Abcam) and GAPDH (1: 1000; ab8245; Abcam), and a horseradish peroxidase conjugated goat anti-rabbit Immunoglobulin G secondary antibody. Then enhanced chemiluminescence reagent (Thermo Fisher Scienti) was applied [35].

### 2.16. Luciferase reporter gene assay

HIPK1 3ʹuntranslated region (UTR) consisting of wild-type (WT) or mutant (MUT) binding sites of miR-30 c-5p was cloned into the pmirGLO vector (Promega), named HIPK1-WT and -MUT. HT22 cells were seeded into 24 well plates at a concentration of 2 × 10^4^ cells/well, and transfection of the cells was with HIPK1-WT and -MUT, and miR-30 c-5p mimic or its NC (RiboBio). Detection of the luciferase activity was via dual luciferase reporter detection system (Promega) [36].

### 2.17. Statistical analysis

SPSS 21.0 (SPSS Inc, Chicago, IL, USA) statistical software was applied for analysis of data. After examination of the kolmogorov smirnov test, the data were normally distributed, expressed as mean ± standard deviation (SD). Application of t test was in the comparison between the two groups, and one-way analysis of variance (ANOVA) in comparison among multiple groups, and Fisher’s least significant difference t test (LSD-t) for pair comparison after ANOVA analysis. *P* was a bilateral test, and *P* < 0.05 was considered statistically significant.

## 3. Results

The study was to explore the beneficial effects of SEV on CIRI in rats. The molecular mechanism of SEV in CIRI was further investigated. Meanwhile, through a series of *in vitro* and *in vivo* experiments, it was found that SEV protected CIRI via modulating the expression of HIPK1 through miR-30 c-5p. Therefore, in the data, the function and mechanism of miR-30 c-5p and HIPK1 in CIRI were first studied, providing new insights into the pathogenesis of CIRI.

### 3.1. SEV reduces CIRI in vivo

**Figure 1.**
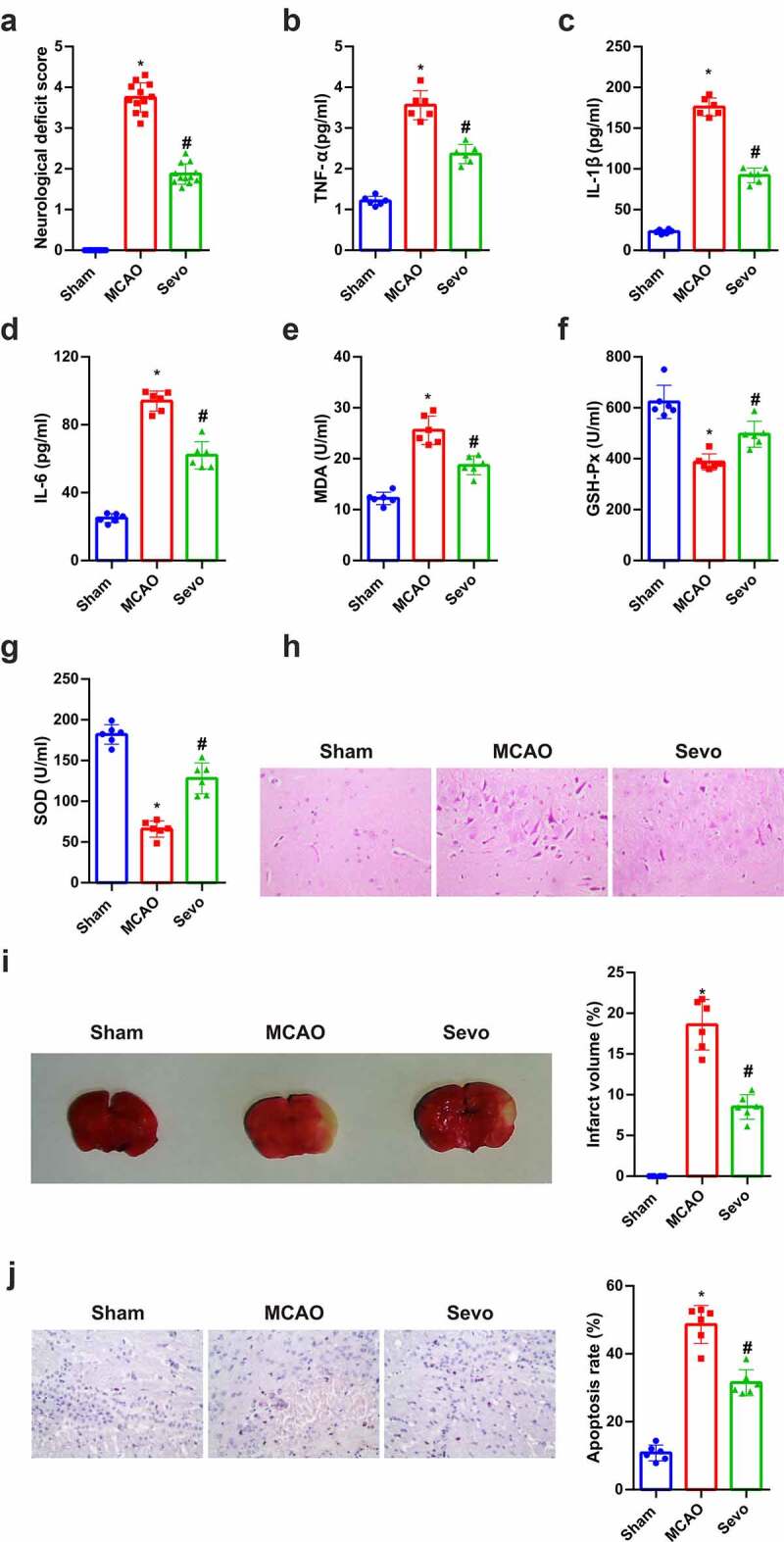
**Amelioration of CIRI via Sev *in vivo.*** (a) Neurological impairment score; (b-d) Inflammation factors TNF-α, IL-1β and IL-6 in rat brain tissue detected via ELISA; (e-g) the content of oxidative stress indexes MDA, SOD, GSH-Px in rat brain tissue detected via ELISA; (h) He staining to observe the pathological damage of brain tissue; (i) TTC staining images to evaluate the cerebral infarction area in each group, and the quantified relative infarct rate to evaluate the effect of SEV on MCAO-induced brain injury; (j) TUNEL staining to detect the apoptosis of brain tissue. (a) n = 12; b-j, n = 6; The data were expressed as mean ± SD; * vs. the Sham, *P* < 0.05; # vs. the MCAO, *P* < 0.05

SEV, a crucial volatile anesthetic, has been affirmed to ameliorate myocardial I/R injury and reduce infarct size [14]. For understanding the effect of SEV on brain tissues of CIRI rats, relevant validation was conducted. Neurological impairment scores were performed in line with Zea-Longa criteria, assuring that in the MCAO the scores were apparently elevated versus the Sham, and distinctly declined after SEV treatment (Figure 1a). Inflammation and oxidative stress are the major pathological mechanisms of CIRI. The anti-CI/RI mechanism of SEV *in vivo* was explored from the above two angles. The data manifested that CIRI rats had inflammation and oxidative stress, and after SEV intervention, TNF-α, IL-1β, IL-6 and MDA were apparently reduced, whereas GSH-Px and SOD were distinctly elevated (Figure 1b-g). The pathological damage of brain tissue was observed (Figure 1h), affirming that in the Sham, the neurons had normal structure and were orderly arranged, with pale red cytoplasm, blue nuclei and clear nucleoli. In addition to necrosis, neurons in the MCAO were disordered, with deeper staining, nuclear membrane rupture, cell structure disappearance, karyopyknosis, hyperchromatic nucleus and lot of lysis. While neurons in the SEV were reduced in swelling, arranged in order, and necrotic cells were declined, and pathological conditions were ameliorated versus the MCAO control. TTC staining manifested that compared with the Sham group, the infarct volume of rats in the MCAO group was clearly elevated, but apparently reduced after SEV inhalation (figure 1i). Moreover, TUNEL staining clarified that the number of apoptotic cells in the MCAO was the highest in the brain tissue of rats, but obviously declined after treatment with SEV (Figure 1j). These experiments indicated that SEV mitigates CIRI *in vivo*.

### 3.2. SEV protects oxygen glucose deprivation/reoxygenation (OGD/R) induced HT22 cell damage

**Figure 2.**
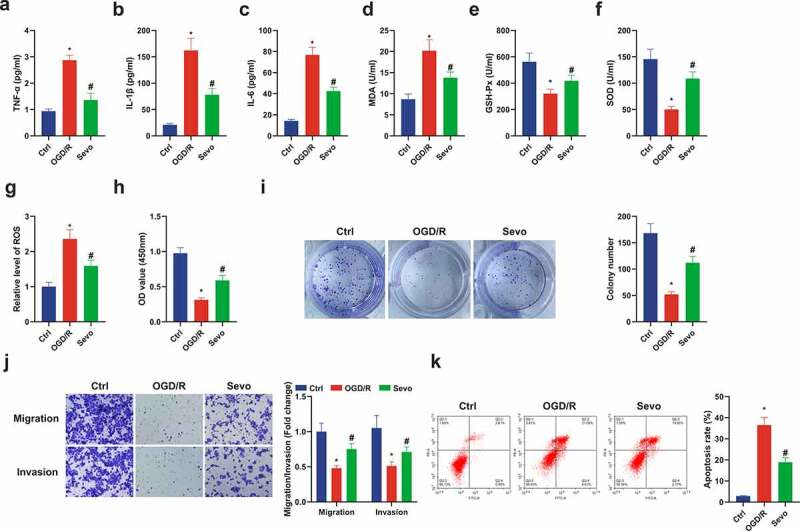
**SEV has a protective influence on Ogd/ R-induced HT22 cell damage**. (a-c) TNF-α, IL-1β and IL-6 in HT22 cells detected by ELISA; (d-f) MDA, SOD, GSH-Px in HT22 cells detected via ELISA; (g) ROS detection in HT22 cells; (h) Cell viability detection via CCK-8; (i) Cell proliferation detected by plate cloning; (j) Transwell to detect cell migration and invasion; (k) Flow cytometry detection of apoptosis. n = 3; The data were expressed as mean ± SD; * vs. the Ctrl, *P* < 0.05; # vs. the Ogd/R, *P* < 0.05

For exploration of the protective mechanism of SEV *in vitro*, establishment of an *in vitro* OGD/R model was also presented. It was manifested the similar inflammatory and oxidative stress responses in the *in vitro* OGD/R mode. In the cell supernatant in the SEV intervention group, apparent decline in inflammatory factors and MDA, and elevation in GSH-Px and SOD were revealed (Figure 2a-f). Former studies have found that the main cause of oxidative stress is the strengthening ROS [37]. Therefore, ROS levels were detected in HT22 cells, demonstrating that Ogd/R up-regulated ROS levels in HT22 cells, which were apparently reduced after SEV treatment (figure 2g). Moreover, SEV was available to improve cell viability, migration and invasion and decline apoptosis after OGD/R treatment (Figure 2h-k). In short, SEV ameliorated inflammation and oxidative stress *in vivo* and *in vitro*, thereby repressing apoptosis and protecting Ogd/R-induced HT22 cell damage.

### 3.3. SEV modulates miR-30 c-5p and HIPK1 in CIRI

**Figure 3.**
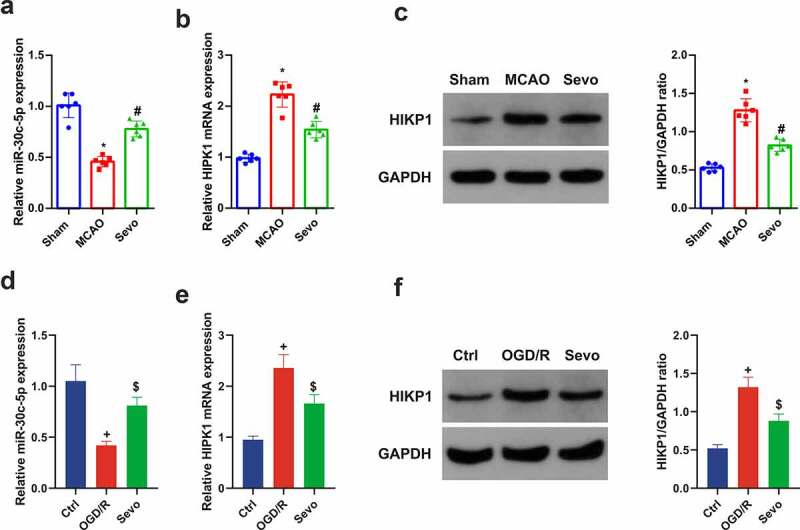
**SEV controls miR-30 c-5p and HIPK1 *in vitro* and *vivo* models of CIRI**. (a) Apparent decline of miR-30 c-5p *in vivo* model of CIRI, while restoration of miR-30 c-5p via SEV; (b-c) Clear elevation of HIPK1 *in vivo* model of CIRI, while silence of HIPK1 via SEV; (d) Obvious depression of miR-30 c-5p *in vitro* Ogd/R model, while elevation via Sev; (e-f) Up-regulation of HIPK1 *in vitro* Ogd/R model, while silence via SEV; (a-c) n = 6, (d-f) n = 3; The data were expressed as mean ± SD; * vs. the Sham, *P* < 0.05; # vs. the MCAO, *P* < 0.05; + vs. the Ctrl, *P* < 0.05; $ vs. the Ogd/R, *P* < 0.05

For exploration of the molecular mechanism of the protection of SEV on CIRI *in vitro* and *vivo*, the influence of SEV on mir-30 c-5p and HIPK1 was discussed. It was affirmed clear repression of miR-30 c-5p and elevation of HIPK1 in CIRI *in vitro* and *vivo* models, whereas distinct amelioration of miR-30 c-5p and HIPK1 with different degrees were manifested after SEV intervention. In brief, it was suggested that the protective mechanism of SEV against CIRI may be implicated with the regulation of miR-30 c-5p and HIPK1 (Figure 3).

### 3.4. Down-regulated miR-30 c-5p eliminates the protection of SEV in vivo

**Figure 4.**
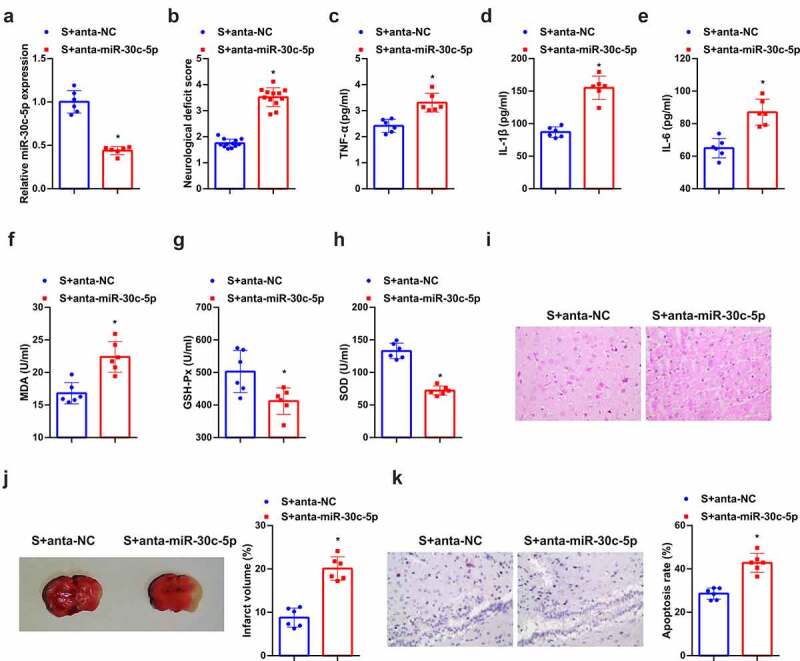
**Knockdown miR-30 c-5p can remove the protection of SEV *in vivo.*** (a) After injection of miR-30 c-5p antagonist, the qPCR detection of miR-30 c-5p in rat brain tissue; (b) Neurological impairment score; (c-e) TNF-α, IL-1β and IL-6 in rat brain tissue detected by ELISA; (f-h) MDA, SOD and Gsh-Px in rat brain tissue detected by ELISA; (i) He staining to observe the pathological damage of brain tissue; (j) TTC staining images to evaluate the cerebral infarction area in each group of rats, and the quantified relative infarct rate to evaluate the effect of repressive miR-30 c-5p on MCAO-induced brain injury; (k) TUNEL staining to detect the apoptosis of brain tissue. (b) n = 12; (a) & (c-k), n = 6; The data were expressed as mean ± SD; * vs. the SEV + antagomir nc, *P* < 0.05

Further discussion of the function of miR-30 c-5p was in the protective mechanism of SEV *in vivo*, distinct repression of miR-30 c-5p was affirmed (Figure 4a). It was assured augmented neurological deficit score in the anta-miR-30 c-5p versus the SEV+ anta-NC (Figure 4b). In the meantime, depressed miR-30 c-5p also reversed the therapeutic influence of SEV on inflammation and oxidative stress (Figure 4c-h). As manifested in Figure 4i, under light microscope, neurons in the SEV group had light swelling, orderly arrangement, and fewer necrosis cells. Knockdown miR-30 c-5p caused disordered arrangement of neurons in rat brain tissue, deeper staining, nuclear membrane rupture, cell structure loss, nuclear pyknosis, hyperchromatic nuclei, and lots of lysis. In addition, after reduced miR-30 c-5p, the infarct volume of rat brain tissue was also apparently elevated (Figure 4j). Tunel staining affirmed fewer apoptotic cells in the brain tissue of rats treated with SEV, and apparently elevated ones after the down-regulation of miR-30 c-5p (Figure 4k). Briefly, knockdown miR-30 c-5p could eliminate the protection of SEV *in vivo*.

### 3.5. Suppressive miR-30 c-5p removes the protection of SEV in vitro

**Figure 5.**
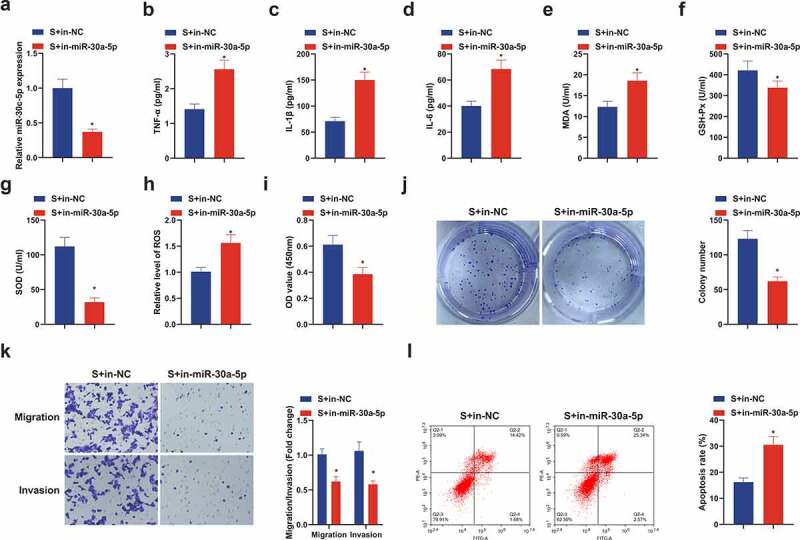
**Knockdown miR-30 c-5p can remove the protection of SEV *in vitro.*** (a) After transfection with miR-30 c-5p inhibitor, the qPCR detection of miR-30 c-5p in HT22 cells; (b-d) TNF-α, IL-1β and IL-6 in HT22 cells detected by ELISA; (e-g) MDA, SOD and Gsh-Px in HT22 cells detected by ELISA; (h) ROS detection in HT22 cells; (i) Cell viability detection via CCK-8; (j) Cell proliferation detected by plate cloning; (k) Transwell to detect cell migration and invasion; (l) Flow cytometry detection of apoptosis. n = 3; The data were expressed as mean ± SD; * vs. the S + in-NC, *P* < 0.05

Next, the role of miR-30 c-5p was also discussed in the protective mechanism of SEV *in vitro*, and acquisition of depressive miR-30 c-5p was manifested (Figure 5a). Elisa results manifested that repressive miR-30 c-5p obviously reversed the depressive effect of SEV on TNF-α, IL-1β and IL-6 expression in CIRI rat model (Figure 5b-d), and augmented MDA, but restrained GSH-Px and SOD were revealed (Figure 5e-g). Meanwhile, ROS was up-regulated in the S + in-miR-30 c-5p versus the S + in-NC (Figure 5h). Moreover, in-miR-30 c-5p also reversed the promotion of SEV on cell viability, migration and invasion and the repression of apoptosis (Figure 5i-l). In sum, curbing miR-30 c-5p eliminates the protection of SEV on OGD/R-induced HT22 cells.

### 3.6. MIR-30 c-5p targets HIPK1 in vitro and negatively regulates its expression

**Figure 6.**
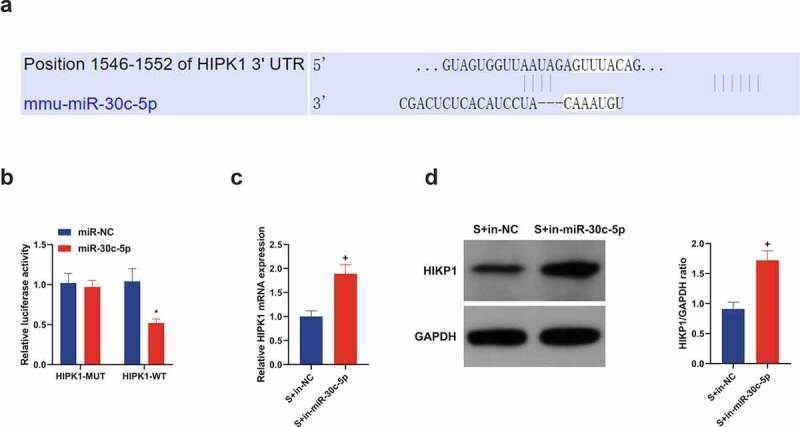
**miR-30 c-5p *in vitro* targets HIPK1 and negatively controls its expression**. (a) Through bioinformatics analysis forecasted the binding site of miR-30 c-5p with HIPK1 (http://starbase.sysu.edu.cn/); (b) In HT22 cells transfected with miR-30 c-5p mimic, the combination of miR-30 c-5p and HIPK1 verified by luciferase reporter gene assay; (c) RT-qPCR detection of HIPK1 in cells introduced with in-miR-30 c-5p; (d) Western blot detection of HIPK1 in cells introduced with in-miR-30 c-5p; n = 3; The data were expressed as mean ± SD; * vs. the miR-Nc, *P* < 0.05; + vs. the S + in-NC, *P* < 0.05

Through bioinformatics analysis was forecasted the binding site of miR-30 c-5p with HIPK1 (Figure 6a), which was then validated. As clarified in Figure 6b, the luciferase activity of pmirglo-HIPK1-WT was restrained, while that of MUT affirmed no obvious change in HT22 cells introduced with miR-30 c-5p mimic. Additionally, in the OGD/R cell model, in-miR-30 c-5p transfection triggered strengthened HIPK1 in HT22 cells versus in-NC transfection, and western blot analysis manifested similar findings (Figure 6c,d). All in all, miR-30 c-5p targets HIPK1.

### 3.7. Repressive HIPK1 reverses the effect of reduced miR-30 c-5p on CIRI in vitro

**Figure 7.**
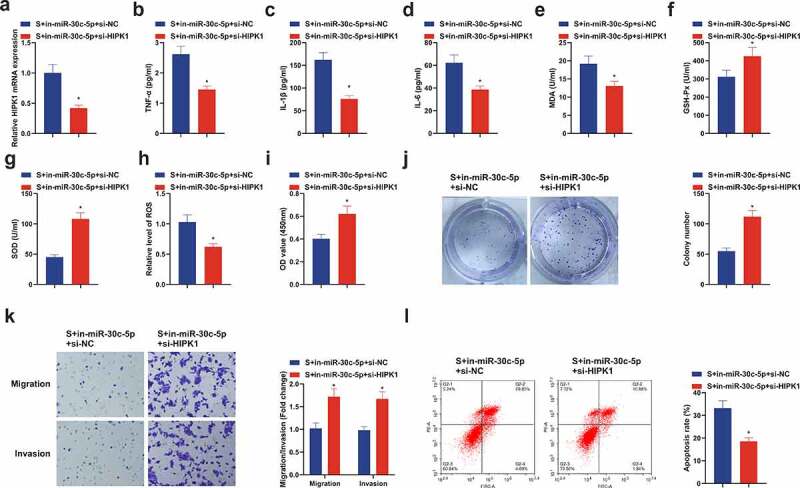
**Repressive HIPK1 reverses the effect of reduced miR-30 c-5p on CIRI *in vitro.*** (a) After transfection of si-HIPK1, the qPCR detection of HIPK1 in HT22 cells; (b-d) TNf-α, Il-1β and IL-6 in HT22 cells detected by ELISA; (e-g) MDA, SOD and GSH-Px in HT22 cells detected by ELISA; (h) ROS detection in HT22 cells; (i) Cell viability detection via CCK-8; (j) Cell proliferation detected by plate cloning; (k) Cell migration and invasion detection via Transwell; (l) Flow cytometry detection of apoptosis. n = 3; The data were expressed as mean ± SD; * vs. the S + in-miR-30 c-5p + si-NC, *P* < 0.05

Further research of the regulatory role of HIPK1, the downstream target gene of miR-30 c-5p, was on CIRI, then transfection of in-miR-30 c-5p + si-HIPK1 was implemented in HT22 cells (Figure 7a), clarifying the depression of inflammatory factors, MDA and ROS, and elevation of Gsh-Px and SOD in the S + in-miR-30 c-5p + si-HIPK1 (Figure 7b-h). What’s more, si-HIPK1 also reversed the suppressive effect of in-miR-30 c-5p on cell growth (Figure 7i-l). In short, repressive HIPK1 reverses the effect of reduced miR-30 c-5p on CIRI *in vitro*.

## 4. Discussion

In this study, SEV was available to ameliorate CIRI by regulating oxidative stress, inflammatory response, cell proliferation, apoptosis, etc. Other experiments have manifested that SEV can repress the decline of miR-30 c-5p stimulated via I/R, thereby restraining HIPK1, namely, SEV can mitigate CIRI through the miR-30 c-5p/HIPK1 axis.

CIRI is a crucial pathological factor exacerbating brain dysfunction, leading to unpleasing treatment and prognosis of ischemic stroke [38]. CIRI, a pathological phenomenon, refers to the recovery of blood flow supply after being blocked for a short time during brain surgery and is implicated with augmented brain dysfunction and structural damage [39]. Although CIRI, an extremely familiar traumatic nervous system illness, is linked with surprising mortality and disability rates [40], current treatment approaches are not quite effective. Hence, it is momentous to explore the molecular mechanism of CIRI for developing brand-new treatment strategies or preventive measures for CIRI heart disease. In the meantime, the therapeutic effect of SEV on CIRI has been testified for lots of times [41,42], but the research of its downstream mechanism requires further exploration. In this study, a rat model of CIRI and an *in vitro* model of OGD/R cells were constructed, and the therapeutic effect of SEV on CIRI was originally verified, manifesting the amelioration of oxidative stress, inflammatory response and histopathologic condition, and reduction of apoptosis, and facilitation of cell growth *in vitro*.

Studies have assured that the therapy of diseases via drugs is often achieved through downstream molecular mechanisms. For instance, notoginsenoside R1 protects the injury induced by hypoxia and re-oxygen deprivation via upregulation of miR-132 in H9c2 cells [43], and demedetomidine modulates miR-205-5p via targeting cerebral ischemia/reperfusion HMGB1 for repression of inflammatory response and oxidative stress [44]. It was discovered that SEV is also associated with miR-30 c-5p/HIPK1 axis, embodying that SEV controls miR-30 c-5p and HIPK1 *in vitro* and *vivo* models of CIRI, and restrains the down-regulation of miR-30 c-5p caused via I/R, thereby repressing HIPK1.

miR-30 c-5p, a member of the miRNA family, has been reported as a key mediator of several cardiac abnormalities, and is commonly applied as a therapeutic target for ameliorating myocardial ischemia-reperfusion injury [45]. In addition, studies have clarified that elevated miR-30 c-5p can rescue ox-LDL-induced decreased cell vitality, and elevated apoptosis and oxidative stress, providing a new mechanism for understanding the pathogenesis of atherosclerosis [46]. However, miR-30 c-5p has not been fully studied in cerebral ischemia reperfusion, so miR-30 c-5p was selected as the research object, to explore whether it has the same effect in cerebral ischemia reperfusion. In the study, it was testified the distinct decline of miR-30 c-5p in CIRI. After knockdown of miR-30 c-5p *in vivo* and *vitro* models pretreated by SEV, oxidative stress, inflammatory response and histopathologic condition were aggravated, and apoptotic cells in tissues were strengthened. For the cell model, the degree of cell damage was augmented, with restrained cell growth, manifesting that repressive miR-30 c-5p is available to reverse the influence of SEV pretreatment on neuronal cells.

The miR-30 c-5p-HIPK1 axis was a novel molecular regulatory network of CIRI pathogenesis, which was originally discovered in the study. HIPK1 is a serine/threonine kinase affiliated to the CMGC superfamily. a study has testified that HIPK1, as a target gene of miR-495-3p, takes part in the modulation of the development of acute kidney injury [19]. In this study, it was clarified originally that HIPK1 was apparently upregulated in CIRI and a target gene of miR-30 c-5p. Meanwhile, it was also demonstrated that depressive HIPK1 could reverse the role of reduced miR-30 c-5p, thus participating in *in vitro* and *in vivo* modulation of CIRI. Of course, there are still some shortcomings in this study. For example, only male rats were applied in animal selection. Cell culture was conducted using higher oxygen concentrations relative to brain tissue; in the meantime, the latent clinical efficacy of this axis will be further complemented later as conditions permit.

## 5. Conclusion

All in all, the study demonstrates that SEV is available to ameliorate CIRI, and this effect may be achieved through miR-30 c-5p/HIPK1 axis. These new findings and mechanisms may provide a potential therapeutic approach for CIRI in the future.
